# Artificial Intelligence in Healthcare Competition (Teknofest-2021): Stroke Data Set

**DOI:** 10.5152/eurasianjmed.2022.22096

**Published:** 2022-10-01

**Authors:** Ural Koç, Ebru Akçapınar Sezer, Yaşar Alper Özkaya, Yasin Yarbay, Onur Taydaş, Veysel Atilla Ayyıldız, Hüseyin Alper Kızıloğlu, Uğur Kesimal, İmran Çankaya, Muhammed Said Beşler, Emrah Karakaş, Fatih Karademir, Nihat Barış Sebik, Murat Bahadır, Özgür Sezer, Batuhan Yeşilyurt, Songul Varlı, Erhan Akdoğan, Mustafa Mahir Ülgü, Şuayip Birinci

**Affiliations:** 1 Department of Radiology, Ankara City Hospital, Ankara, Türkiye; 2 Department of Computer Engineering, Artificial Intelligence Division, Hacettepe University, Ankara, Türkiye; 3 Telemedicine and Teleradiology, Simplex IT, Inc, Ankara, Türkiye; 4 General Directorate of Health Information Systems, Ministry of Health, Ankara, Türkiye; 5 Department of Radiology, Sakarya University Faculty of Medicine, Sakarya, Türkiye; 6 Department of Radiology, Isparta Süleyman Demirel University Faculty of Medicine, Isparta, Türkiye; 7 Department of Radiology, GOP University Faculty of Medicine, Tokat, Türkiye; 8 Department of Radiology, Ankara Training and Research Hospital, Ankara, Türkiye; 9 Department of Radiology, Van Training and Research Hospital, Van, Türkiye; 10 Health Institutes of Türkiye, İstanbul, Türkiye; 11 Department of Computer Engineering, Konya Technical University Faculty of Engineering and Natural Sciences, Konya, Türkiye; 12 Department of Computer Engineering, Yıldız Technical University, İstanbul, Türkiye; 13 Department of Mechatronics Engineering, Yıldız Technical University Faculty of Mechanical Engineering, İstanbul, Türkiye; 14 Ministry of Health, Ankara, Türkiye

**Keywords:** Computer vision, stroke, data set, artificial intelligence, competition

## Abstract

**Objective:**

The artificial intelligence competition in healthcare was organized for the first time at the annual aviation, space, and technology festival (TEKNOFEST), Istanbul/Türkiye, in September 2021. In this article, the data set preparation and competition processes were explained in detail; the anonymized and annotated data set is also provided via official website for further research.

**Materials and Methods:**

Data set recorded over the period covering 2019 and 2020 were centrally screened from the e-Pulse and Teleradiology System of the Republic of Türkiye, Ministry of Health using various codes and filtering criteria. The data set was anonymized. The data set was prepared, pooled, curated, and annotated by 7 radiologists. The training data set was shared with the teams via a dedicated file transfer protocol server, which could be accessed using private usernames and passwords given to the teams under a non-disclosure agreement signed by the representative of each team.

**Results:**

The competition consisted of 2 stages. In the first stage, teams were given 192 digital imaging and communications in medicine images that belong to 1 of 3 possible categories namely, hemorrhage, ischemic, or non-stroke. Teams were asked to classify each image as either stroke present or absent. In the second stage of the competition, qualifying 36 teams were given 97 digital imaging and communications in medicine images that contained hemorrhage, ischemia, or both lesions. Among the employed methods, Unet and DeepLabv3 were the most frequently observed ones.

**Conclusion:**

Artificial intelligence competitions in healthcare offer good opportunities to collect data reflecting various cases and problems. Especially, annotated data set by domain experts is more valuable.

**Main Points**

- Artificial intelligence (AI) competitions in healthcare offer good opportunities to collect data reflecting various cases and problems.
- Annotated data set will contribute to the new AI model studies.
- Artificial intelligence competitions in healthcare have increased the interoperability of people from different disciplines and educational levels.

## Introduction

TEKNOFEST is an annual aviation, space, and technology festival organized in Türkiye since 2018. With its primary focus on technology, the festival’s scope is being extended every year. Atatürk, the founder and first president of the Republic of Türkiye, showed a critical direction to the young republic by saying, “The future is in the skies.”^1^ With TEKNOFEST, it aims to raise public awareness about technology and aerospace in society and encourages young minds to take roles in these fields. In addition, it is aimed to contribute to the qualified human resources trained in the relevant technology by hosting many activities such as technology competitions, air shows, concerts, talks, and events on various subjects. Technology competitions in various disciplines and categories are organized within the scope of TEKNOFEST. People with many different education and experience levels such as primary school, secondary school, high school, undergraduate postgraduate, and graduate, and entrepreneur-private sector members participated in these competitions individually or in groups. The number of competition categories has increased from 14 in 2018 to 35 in 2021, including intelligent transportation, helicopter design, biotechnology, robotics, flying cars, rockets, and artificial intelligence (AI) in healthcare categories. The AI competition in healthcare was organized for the first time in 2021. The domain of problem in the AI in healthcare competition was “Stroke.”^2^

Hemorrhagic and ischemic stroke cases have an important place among emergency department admissions. While 15% of all strokes in the United States present with hemorrhagic stroke, the other 85% is ischemic stroke.^3^ When evaluating a stroke patient, determining whether the type of stroke is ischemic or hemorrhagic is the most critical clinical point in the follow-up and treatment of the patient since the treatment approaches of these stroke types are entirely different. Because of interventional vascular radiological procedures, thrombolytic and anticoagulant drugs are given to open the occluded vessel in ischemic type stroke, while these drugs are contraindicated in hemorrhagic type stroke.^4^ In hemorrhagic type stroke, treatments are applied to reduce the bleeding (reducing the hypertensive status and reducing the accompanying edema) and to eliminate the causes that cause hemorrhage (closure of the underlying aneurysm). Both radiologists and clinicians should diagnose patients earlier, thus preventing morbidity and mortality from a stroke.

Artificial intelligence is reaching an increasingly widespread use in terms of excluding typical cases in order to reduce the heavy workload and to make a rapid diagnosis of patients with hemorrhage or infarction. Artificial intelligence can eliminate the workload and pressure on emergency departments by excluding typical head computed tomography (CT) scans.^5^ Algorithms that make triage or classifications such as intraparenchymal, intraventricular, extra-axial, or subarachnoid hemorrhages were designed with the help of machine learning (ML) for acute intracranial hemorrhages.^6-8^ Automated segmentation and measurements of hematoma can be achieved with a non-contrast head CT scan.^9-11^ Compared to expert radiologists, AI can achieve similar or better results in detecting early ischemic changes.^12-14^ In non-contrast head CT, the results compatible with diffusion-weighted magnetic resonance imaging (MRI), the reference standard for ischemia, can also be obtained through ML.^15,16^ Artificial intelligence provides many conveniences in stroke management processes at many stages, such as the detection and differentiation of hemorrhagic and ischemic stroke, quantification of the amount of bleeding, segmentation of ischemic core volume, determination of large vessel occlusion, treatment selection, and estimation of prognosis.^17^

In the AI competition in healthcare at the TEKNOFEST, it was asked from the competitors, by using non-contrast CT head images, in the first stage, to develop a model to categorize CT head images as the stroke “absent or present” and in the second stage, to develop a model to detect and segment the areas of “hemorrhage or ischemia” in stroke patients. In this article, we aim to describe the data set and how the data set of this challenge was collected, prepared, pooled, curated, annotated, anonymized, shared, and how the evaluation process was managed in the first and second stages at the competition.

## Materials and Methods

### Timeline

The competitions within the scope of TEKNOFEST 2021 were launched on February 3, 2021. Team applications for participation in the AI in healthcare competition were received until March 15, 2021. Regarding the theme concerning the AI in healthcare competition, a data set preparation team including field experts was formed for the data set preparation process on March 13, 2021. On May 5, 2021, the data set preparation process was completed, and the data set was shared with the teams. The AI in healthcare competition stages were held on September 23 and 24, 2021. The top 3 teams were determined with the final competition held on September 24, 2021.

The data set was anonymized, and the data set preparation was organized by the Republic of Türkiye, Ministry of Health. The study was approved by the Republic of Türkiye, Ministry of Health (November 12, 2021/E-67523305-702.99-7553). Informed consent was waived because of the retrospective anonymous data analysis of national health database survey. 

## Data Overview

### Data Preparation and Collection

First task for the data preparation was establishing a data set preparation team consisting of field experts. Then the number of head CT sections to be labeled and segmented for pre-competition training and testing phases was determined. The most influential parameters on this decision were the remaining time interval for data set sharing and the daily work routine of the domain experts. Pre-competition train and test data sets were required to give opportunities to be ready at the competition time to use their fine-tuned models directly. Accordingly, characteristics of the data set for pre-competition phase were determined as follows: 4400 head CT sections with no signs of acute/hyperacute stroke or within normal limits, 1100 sections with signs of hyperacute/acute ischemic stroke, and 1100 sections with signs of hemorrhagic stroke. A total of 200 head CT sections (130 with no signs of acute/hyperacute stroke or within normal limits, 35 with signs of hemorrhagic stroke, and 35 with signs of hyperacute/acute ischemic stroke) were planned to be used in testing at the first stage of the competition. A further 100 head CT sections (48 with hyperacute/acute ischemic stroke findings, 47 with hemorrhagic stroke findings, and 5 with both findings in the same section or with pathologies that could be included in differential diagnosis) were planned to be used for the second stage of the competition.

For the planned training and test data sets, the data recorded over the period covering 2019 and 2020 were centrally screened from the e-Pulse and Teleradiology System of the Republic of Türkiye, Ministry of Health using various codes and filtering criteria. During this process, a search was conducted using the International Classification of Diseases (ICD-10) codes, diagnostic and interventional procedure codes for radiological imaging and treatment, and keywords and sentence structures included in free-text radiology reports. The codes, filtering rules, keywords, and sentence structures used in the search are shown in Table 1. Considering the data entry times of the ICD-10 codes and the radiological imaging and interventional procedure codes, the “+/−15 days” time filter was used for the search.

### Data Pooling and Curation

As a result of the search and screening process, a total of 67 000 cases were retrieved by the filter of “ICD-10” and “Head CT” criteria. And also, 18 000 satisfied the “Diffusion MRI” criterion, 6500 met the “Thrombectomy for acute stroke,” “Head CT,” “Endovascular cerebral aneurysm treatment,” and “Head CT” criteria. At last, 116 000 cases contained the keywords and sentence structures searched in free-text radiology reports.

In order to reach the head CT cross-section numbers determined initially for the training and testing stages, 2 radiologists with 9 years of experience in the radiology field randomly examined 5000 cases from a large data pool over 2 weeks. Among these cases, 1000 were selected to create a training and test data set pool by considering the following:

The acquisition times of the head CT and diffusion MRI examinations were considered; head CT and diffusion MRI images were examined together in ischemic stroke cases, especially in hyperacute processes; an ischemic stroke was considered acute if the patient presented from 24 hours to 1 week within the stroke onset and hyperacute if within the first 24 hours; diffusion MRI was considered the gold standard method to confirm the acute/hyperacute ischemic stroke processes suspected in head CT; in ischemic stroke cases, care was taken to ensure that the interval between the head CT scan and the diffusion MRI scan was very short (within 1-2 hours); a head CT slice thickness of 2-5 mm was used as a selection criterion; it was confirmed that there was no pathology in the short-long-term follow-up of cases with typical head CT findings; cases with chronic ischemic findings were also added to the group without hyperacute/acute stroke symptoms, as they were seen frequently, it was ensured that findings such as benign calcifications, motion artifacts, and beam hardening artifacts, which are very common in routines of radiological practice but were not subject to competition, were also present in the head CT images.

### Data Annotation

The head CT series of 877 cases, whose images could be accessed and pooled from the web application installed on the Ministry of Health server and accessible from the address “teknofest.saglik.gov.tr,” were labeled, segmented, controlled by 7 radiologists through task sharing, and the slice selection process was completed in 6 weeks.

**Figure 1.**
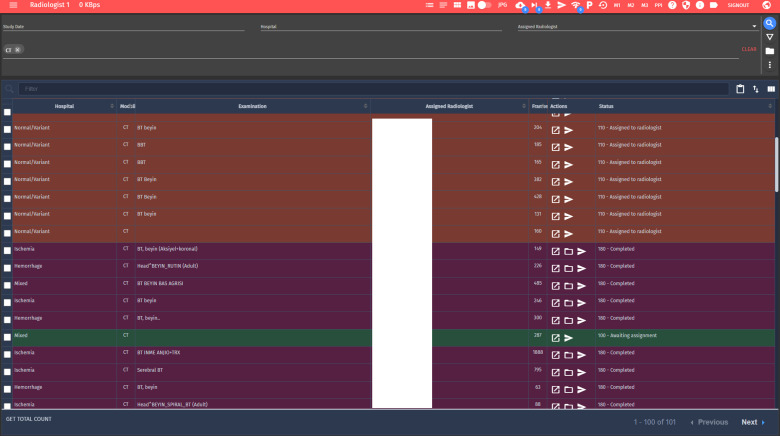
Data annotation and workflow management software, RAD-X Picture Archiving and Communication System Version 2.0 (Simplex IT Inc.-Ankara-Türkiye).

RAD-X Picture Archiving and Communication System (PACS) Version 2.0 developed by Simplex IT Inc.-Ankara-Türkiye (https://simplexbt.com/) was used for data annotation and workflow management. All studies selected for labeling were first imported to RAD-X PACS Server using the built-in data importing function. The software automatically created labeling work lists from imported studies, as seen in Figure 1. Users can search work list items based on the assigned radiologist workflow status (such as ready for labeling, labeling assigned to radiologist, labeling control assigned to radiologist, or labeling completed). Authorized users can create labeling tasks for themselves or other radiologists using this window. Such task assignments prevented radiologists from working on the same study.

**Figure 2.**
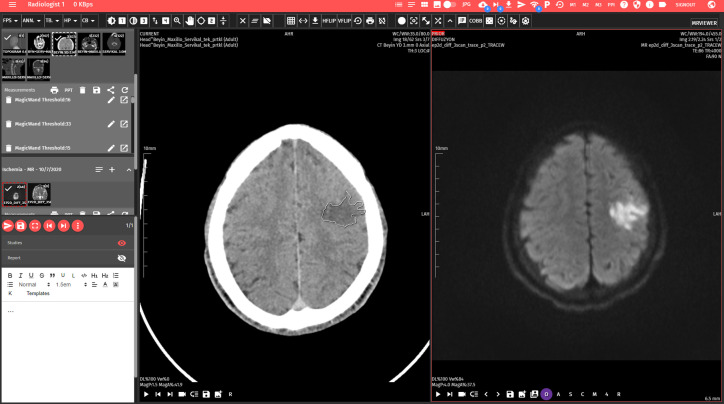
Data viewing and segmentation window.

A labeling and segmentation task can be started by double-clicking on the work list item, which opens an image viewing, labeling, and segmenting window, as shown in Figure 2. The imaging window provides common functions found in most radiological workstations. For image annotation, the following functions have been widely used:

*Study comparison mode: *Allowed the radiologists to compare images of the patient from different modalities. This function eased the labeling of ischemic strokes on CT images by providing spatially synchronized MR slices in the same window.

*Window leveling:* Allowed the radiologists to maximize the contrast between the lesion and surrounding area.

*Zoom/pan:* Allowed more precise annotation of smaller lesions.

*Free hand region selection:* Allowed selection of the boundaries of a lesion manually. 

*Automated region selection:* Allowed selection of the boundaries of a lesion automatically using the watershed algorithm. The threshold value for the algorithm can be set interactively using the mouse.

The users could save their progress at any time before finalizing the annotation task for a given study. Controller radiologists could delete an annotation created by previous user or modify the contours of these annotations. An online education for the use of PACS and annotation tool was given by Y.A.O (PACS and annotation tool provider) to radiologists before the annotation process.

A radiologist who took part in the pooling process and had 9 years of experience in the radiology field assigned the head CT series of the patients to constitute the stroke-present (hyperacute/acute and hemorrhagic) group to 4 radiologists (with 11, 9, 7, and 7 years of experience in the field of radiology, respectively). First, the sections of the hemorrhagic stroke cases and then those of the ischemic stroke cases were segmented. All the sections with pathologies in the head CT sections were segmented. The 4 radiologists were divided into 2 groups and paired. Thus, segmentation undertaken by 1 radiologist was checked by another radiologist. In addition, during the selection of the segmented head CT sections for training and testing, the final control was performed by 2 radiologists with 9 years of experience. Non-consecutive sections were randomly selected from these CT series. 

**Figure 3.**
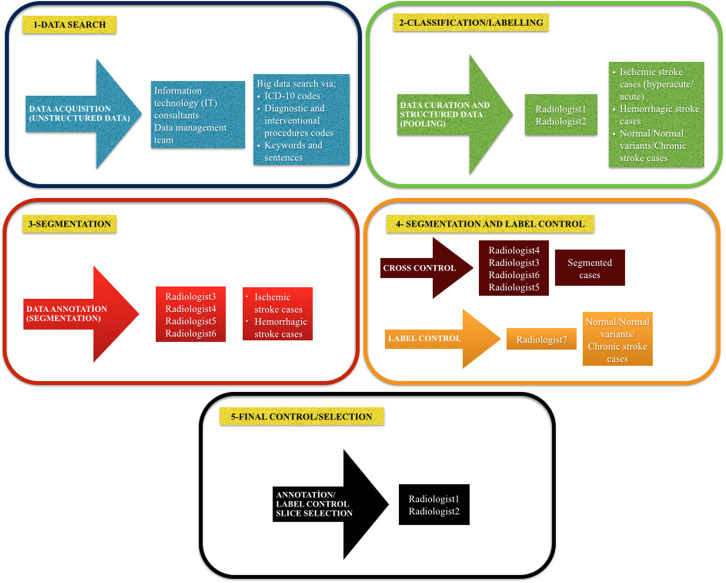
Radiologist task sharing and data set annotation methodology.

The head CT series of the patients in the stroke-absent and chronic ischemic findings group were examined in detail during the pooling stage and reviewed by a radiologist with 4 years of experience before selecting sections. As in the stroke-present group, a final check was performed by 2 radiologists with 9 years of experience. Non-consecutive images were randomly selected from these CT series. The radiologist workflow is summarized in Figure 3.

### Data Anonymization and Sharing

After completing all labeling tasks, annotated images from all studies were moved into 3 data sets for training, competition stage 1, and competition stage 2. The images on each data set were anonymized, and masks were generated for each image. All digital imaging and communications in medicine (DICOM) tags were removed during anonymization except the following ones in Table 2.

In order to create the data set, 877 CT and 230 MRI studies belonging to 819 unique cases were collected. The number of images in the training and competition data sets is given in Table 3. The training data set consisted of 6651 images where 4427 images contained no evidence of stroke, chronic ischemic findings, or only normal findings; 1131 images contained hyperacute/acute ischemic stroke findings, and 1093 images contained hemorrhagic stroke findings. Each image was given a unique id, and for each image, 4 files were generated in 4 different folders (Table 4). Digital imaging and communications in medicine files and the mask files were intended for model training. On the other hand, portable network graphics (PNG) versions of the DICOM files and the overlay images were generated to assist the teams to make the content of the data more understandable.

The training data set was shared with the teams via a dedicated file transfer protocol server, which could be accessed using private usernames and passwords given to the teams under a non-disclosure agreement signed by the representative of each team. Competition data sets consisting of 192 images for stage 1 and 97 images for stage 2 were preserved, and these images were provided to the teams at the beginning of each stage in competition time. Eight images for stage 1 and 3 images for stage 2 were excluded from the test data set due to image properties (non-standard matrix size).

### Evaluation Criteria

The competition consisted of 2 stages. In the first stage, teams were given 192 DICOM images that belong to 1 of 3 possible categories (hemorrhage, ischemic, or non-stroke). Teams were asked to classify each image as either stroke present or absent. Results were submitted as a comma-separated values (CSV) file, which contained the estimation of the models (0 for stroke absent and 1 for stroke present) for all images. The F_1_ score [2*(precision*recall/precision + recall)] was then calculated for every team, and teams with an F_1_ score lower than 0.75 were eliminated. 

In the second stage of the competition, qualifying teams were given 97 DICOM images that contained hemorrhage, ischemia, or both lesions. Teams were asked to generate 97 PNG images that contained pixels with values of 2 for hemorrhage lesions, 1 for ischemic lesions, and 0 for other areas. For evaluation, the intersection over union (IoU) value was calculated for each image by comparing submitted results with the images generated by the radiologists (ground truth). Since the boundaries of the lesions cannot be specified with perfect precision even by the most expert radiologists, intersections were calculated between the submitted image and the dilated version of the ground truth image, a 3 × 3 kernel. Similarly, unions were calculated between the submitted image and the eroded version of the ground truth image with a 3 × 3 kernel. In order to keep the pixel classes during erosion and dilation, ground truth images were first split into 2 images containing only values of either 1 or 2. After erosion and dilation of these 2 images, results were merged to obtain dilated and eroded versions of the ground truth images. Pseudocode of the function that calculates the score for a prediction mask (predicted) submitted by a user is given below. Here, the groundTruth is the mask image generated by the radiologists which is accepted as the ground truth.

*Score (groundTruth, predicted):*

*set mask1(i, j) to 1 where (groundTruth == 1), 0 otherwise // first-class-only mask*

*set mask2(i, j) to 1 where (groundTruth == 2), 0 otherwise // second-class-only mask*

*   erosion1 = erode(mask1) // Binary image erosion with a 3 *×* 3 kernel filled with 1’s, 1 iteration*

*   dilation1 = dilation(mask1) // Binary image dilation with a 3 *×* 3 kernel filled with 1’s, 1 iteration*

*erosion2 = erode(mask2)*

*dilation2 = dilation(mask2)*

*// Merge erosion1 and erosion1*

*set erodedGroundTruth(i, j) to 1 where erosion1(i, j) == 1, to 0 otherwise*

*set erodedGroundTruth(i, j) to 1 where erosion2(i, j) == 1, to 0 otherwise*

*// Merge dilated1 and dilated2*

*set dilatedGroundTruth(i, j) to 1 where dilated1(i, j) == 1, to 0 otherwise*

*set dilatedGroundTruth(i, j) to 1 where dilated2(i, j) == 1, to 0 otherwise*

*set intersection(i, j) to 1 where (dilatedGroundTruth(i, j) == predicted(i, j) and dilatedGroundTruth(i,j) != 0), to 0 otherwise*

*set union(i, j) to 1 where (erodedGroundTruth(i, j) != 0 or predictedMask(i, j) != 0), to 0 otherwise*

*intersectionCount = nonZeroElementCount(intersection)*

*unionCount = nonZeroElementCount(union)*

*score = intersectionCount/ unionCOunt*

*return score*

The obtained IoU scores of the teams were sorted for every image, and the team with the highest IoU score was given a score of 36. The team with the second-highest IoU score was given a score of 35 and so on. Teams having 0 IoU score for an image were all given the same score that is one less than the score of the team having the lowest IoU score greater than 0. So for an image where only 10 teams obtained an IoU score of greater than 0, first 10 teams got a score of 36-27 and the remaining 26 teams got a score of 26. Overall score of each team was calculated by adding their scores from each image. Theoretical maximum score was 3492 (if a team gets the highest IoU scores for all images) and minimum score was 97 (where a team gets the lowest IoU score (greater than 0) for all images).

## Results

**Figure 4.**
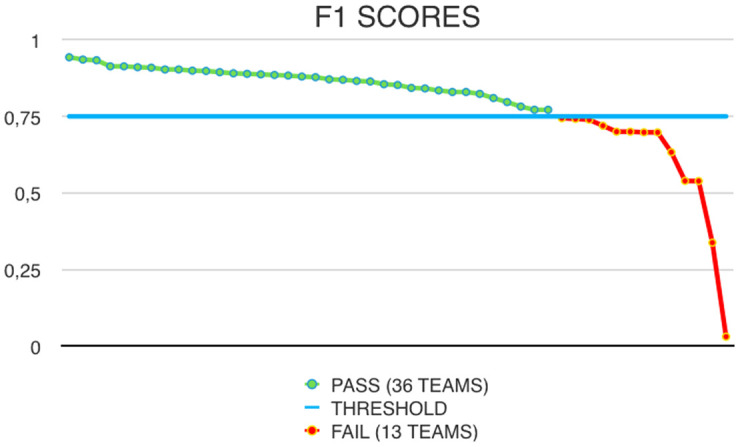
The F1 scores for 49 teams.

**Figure 5.**
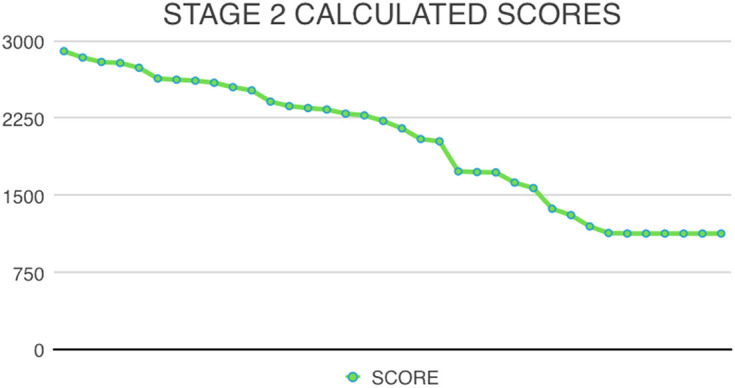
The IoU scores of the 36 teams.

**Figure 6.**
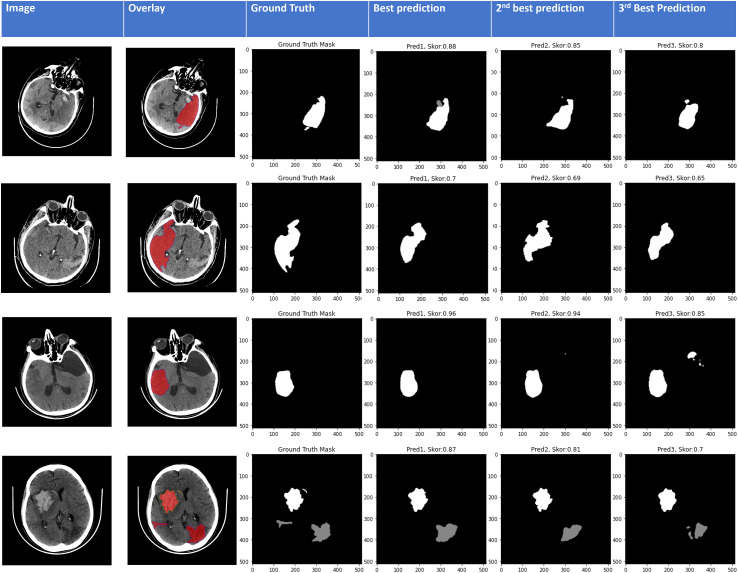
Top 3 IoU scores for 4 images (misleading images). First row CT image consisted of both ischemic core and aneurysm ipsilaterally, second row CT image had both ischemic core and vascular anomaly contralaterally, third row CT image had arachnoid cyst and ischemic core contralaterally, and fourth row CT image had both ischemic cores and hemorrhagic areas. CT, computed tomography.

**Figure 7.**
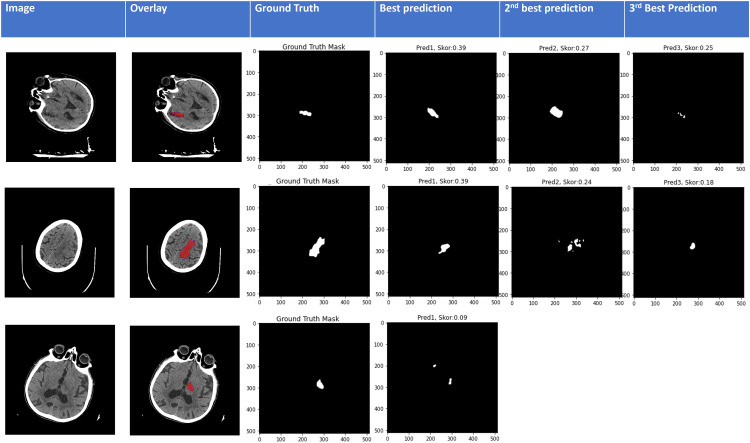
Top IoU scores of 3 images were demonstrated. All of the images are of the hyperacute ischemic stroke cases. Imaging findings that are hard to catch, even for the radiologist.

Teams who had enough scores to attend the competition were carefully selected. While the selection process, 2 different reports were expected from them. First one was about what they would like to do and the second one was asked to understand how their plans go well and what their performance situation is. Approximately, 10% of teams reached the final competition, and demographic data of the team members participating in the competition are presented in Table 5. The F1 scores for 49 teams are shown in Figure 4. Thirty-six teams qualified for the second stage. The IoU scores of teams are given in Figure 5. The ground truth masks and the best predictions for 7 images in the second stage are shown in Figures 6 and 7.

In competition, successful results have been achieved and the mean of the top 3 IoU scores was 0.785. It can be assumed as the value given for the researchers for further studies. 

**Figure 8.**
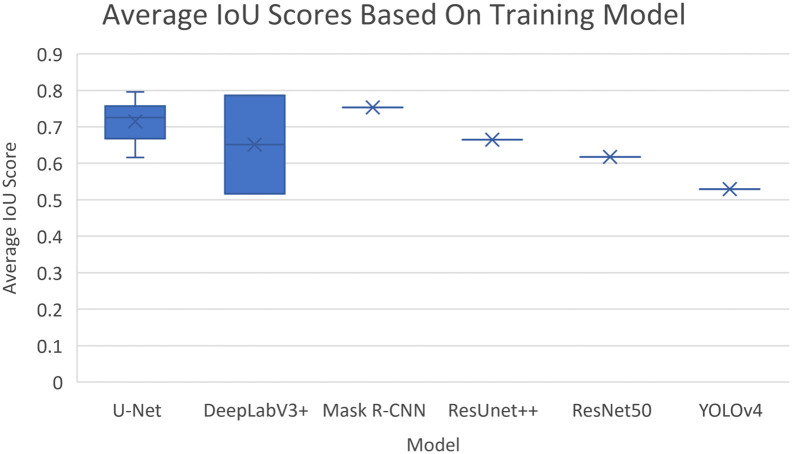
Average IoU scores based on training model.

While the competition, it is observed that most of the proposed models were developed with the help of pre-trained models. Unet and DeepLabv3 were the most frequently employed architectures (Figure 8). In fact, this type of architecture selection preference is quite plausible because each of them has been proposed for either image segmentation or medical image segmentation. Unet takes its name from its U-shaped architecture and deserves attention because of less training data requirement. In the first part of U, there is a convolutional network including repeated convolutions followed by a rectified linear unit and a max-pooling operation. In the second part of U, there is a sequence of up-convolutions and concatenations with high-resolution features from the first U-part. Especially, the boundaries of the segments become more precise. DeepLab is a semantic segmentation architecture that uses dilated convolutions and bilinear interpolation. In DeepLab, fully connected Convolutional random fields are used to constitute the segments. DeepLabv2 built on DeepLab with a spatial pyramid pooling scheme is a solution for the different sizes of the same objects and the last version DeepLabv3 improved DeepLabv2 for the problem of segmenting objects at different scales by cascading convolutions.

## Discussion

Technology and space developments are overgrowing, and hundreds of national–international congresses, fairs, festivals, competitions, and conferences are held every year. The World Science Festival is definitely one of the largest and the most celebrated annual science festival.^18^ In addition, Edinburgh International Science Festival, International Science Festival Gothenburg, British Science Festival, Wide Science Festival, and TEKNOFEST can be given as examples of other festivals that attract worldwide attention. TEKNOFEST draws attention as an organization that is proliferating and trying to take its place in the international arena. 

In recent years, there has been a great interest in AI in the field of radiology. Radiological Society of North America (RSNA) organizes image-based AI competitions and tries to move forward by focusing on problem-oriented solutions with the experiences gained after the competitions.^19^ The dialogue between radiologists, engineers, and data scientists has increased in AI competitions in radiology hosted by RSNA for the last 4 years. These competitions have been shown to be an important event to tackle problems collaboratively.^19^ Radiological Society of North America has hosted several public AI competitions to promote AI-related research in radiology. The competitions focused on estimating the age of pediatric patients based on hand radiographs in 2017, pneumonia detection on chest x-rays in 2018, intracranial hemorrhage detection on brain CTs in 2019, pulmonary embolism detection on chest CTs in 2020, and lastly, coronavirus disease 2019 on radiographs and brain tumor on brain MRIs in 2021.^20^

The AI in healthcare competition at TEKNOFEST was organized for the first time in 2021, carried out by the Republic of Türkiye, Ministry of Health and Health Institutes of Türkiye (TUSEB). It aimed to raise awareness of AI in healthcare and awareness of the stroke problem in the health field. Hemorrhagic and ischemic stroke is a common disease in society, and its incidence increases with age. Early diagnosis, grouping, and treatment of this disease are life-saving. In case of delay in diagnosis and treatment, permanent sequelae and even death can be seen. In this case, rapid and accurate guidance of CT in stroke patients is vital as the first step in patient management. Distinguishing the stroke patient from the typical patient, determining the type of stroke, and showing the brain region involved in the stroke with the AI models that we hope to be developed. The speed of the radiologist will increase, and the margin of error will decrease. Patients, emergency physicians, neurologists, neurosurgeons, and diagnostic and interventional radiologists dealing with stroke will lose less time in the diagnosis phase and will be able to switch to the treatment process.

Data set acquisition and experimentation with different data sets in ML studies are critical. At this point, as examples, the MIMIC III and IV data sets^21^ used in many intensive care studies and the ECGView data set^22^ used in electrocardiogram classification have a significant impact on ML studies. Anatomical Tracings of Lesions After Stroke (ATLAS)^23^ is an open-source data set that consists of 304 T1-weighted MRIs with manually segmented lesions and metadata. For each MRI, brain lesions were identified, and masks were manually drawn on each brain by using the open-source tool for brain imaging visualization and defining volumes of interest. After prepossessing and normalization procedures, 229 MRIs have been released as ATLASv1.1. In PhysioNet, CT images of hemorrhage that were segmented and masked manually have been shared.^24,25^ This data set consists of 82 CT scans, including 36 scans with 5 mm slice-thickness for each patient with intracranial hemorrhage of various types. Exactly 2 radiologists studied each slice of the non-contrast CT scans, and they also delineated the regions in each slice without the patients’ clinical history. As a result, 2500 brain and 2500 bone window images are presented in the form of gray-scale 650 × 650 images (jpg format), and white regions mean the annotated as the hemorrhage. This data set is closer to the data set presented here from the perspective of segmentation, annotation, and masking. However, the presented data set has the focus on brain stroke segmentation as ischemic, hemorrhage, or neutral. For the ischemic lesions, the data set shared while ischemic stroke lesion segmentation 2018 challenge is a good comparative example.^26^ Ischemic stroke lesion segmentation challenges started in 2015 to provide a platform for a fair and direct comparison of automated methods for stroke imaging, and the first public acute stroke data set using CT images was shared in 2018. In that challenge, non-contrast CT imaging has been selected as the data source and the segmentation of ischemic stroke lesion core from acute CT scans, taken within 8 hours of stroke onset. The provided data set includes 94 labeled training images and 62 unlabeled testing images. The images were acquired as slabs with a variable number of axial slices, ranging from 2 to 22 depending on the patient, with 5 mm spacing and a resolution of 256 × 256. The provided gold standard was manually drawn on CT scans. 

When we consider the properties of the presented data set (masks were manually created, existing labels are ischemic, hemorrhage, and other area) and data size, it can be concluded that this data set has the adequate number of instances for the ML or deep learning modeling research, and it contributed the brain stroke subdomain by presenting new classification focus.

The diversity and multiplicity of data sets directly affect the sophistication and maturity of ML models to be made in that field. Therefore, data sets that include human effort while creating the gold standard by field experts are valuable. In this competition, the radiologist took part voluntarily in preparing the data set. Labeling, especially segmentation, is time-consuming and expensive.^27^ Radiologists cannot spare much time for data set preparation and annotation for AI in daily routine due to the low number of radiologists in the country, lack of free time, and high demand for radiological examination. Therefore, annotation tools need to be more practical. Annotating fine or rough detail in the cross-sectional images changes the labeling or segmentation time. For this competition, each radiologist allocated 45 minutes per case in some cases. The data set was created with data from 173 different devices and 6 different vendors. Thus, diversity was achieved. Imaging biobanks repositories are beneficial and critical for training, testing, or validating AI models.^28^ Domain experts make annotated data sets, and data diversity is precious to develop the field of AI in healthcare and allow competing team members to show and improve themselves in the AI ecosystem.

The communication and collaborative work between radiologists and data scientists or engineers is a crucial step for success in developing a radiological AI solution.^29^ In this manner, this competition has increased the interoperability of people from different disciplines and educational levels. It is observed that the mean age was 26.06, and the male gender was dominant in this competition. There was more participation at the undergraduate level than the graduate level. The number of team members from the medical field is 5% among the total participants. Most of them were medical students. It can be deduced that the new generation of medical students is more interested in AI than medical doctors or specialists. There should be greater participation from the medical field as a team member. Opportunities and platforms should be created as advertisements for AI competitions should reach more people. People who work in different disciplines and who are willing to form a team in these AI competitions should be able to find each other.

The most important limitation of this study was that the data set preparation process was short. The other limitations were finding domain experts to label the data and their busy workflow. 

In conclusion, AI competitions and open access annotated data sets will lead to the advancement of AI in medicine and collaboration of the different disciplines. The data set can be reached by http://acikveri.saglik.gov.tr.

## Figures and Tables

**Table 1.** The Codes, Filtering Rules, Keywords, and Sentence Structures Used in the Data Search Are Shown

**ICD-10 codes**	**Procedure Codes for Diagnostic Imaging and Interventional Treatment**
I63.9- Cerebral infarction, unspecified	803.900- Head CT
I61.3- Intracerebral hemorrhage, in the brain stem	803.910- Head CT
I62.9- Intracranial hemorrhage (non-traumatic), unspecified	804.220- Diffusion-weighted MRI
I60.9- Subarachnoid hemorrhage, unspecified	802.891- Thrombectomy for acute stroke
I61.9- Intracerebral hemorrhage, unspecified	802.760- Endovascular cerebral aneurysm treatment
I61.5- Intracerebral hemorrhage, unspecified	
I60.7- Subarachnoid hemorrhage, originating from intracranial arteries, unspecified	
**Filtering criteria**	**Keywords and sentence structure queries in free-text radiology reports**
“ICD-10 code” **and** “Head CT code” **or** “Diffusion MRI”	“Normal head CT”
“Thrombectomy for acute stroke” **and** “Head CT code” **or** “Diffusion MRI”	“Head CT examination within normal limits”
“Endovascular cerebral aneurysm treatment” **and** “Head CT code”	“No acute cranial CT finding was detected”

**Table 2.** Non-removed Digital Imaging and Communications in Medicine Tags During Anonymization Are Shown

**Tag**	**Description**	**Tag**	**Description**
**(0002,0000)**	FileMetaInformationGroupLength	**(0028,0101)**	BitsStored
**(0002,0003)**	MediaStorageSOPInstanceUID	**(0028,0102)**	HighBit
**(0002,0010)**	TransferSyntaxUID	**(0028,0103)**	PixelRepresentation
**(0018,0050)**	SliceThickness	**(0028,0120)**	PixelPaddingValue
**(0028,0002)**	SamplesPerPixel	**(0028,1050)**	WindowCenter
**(0028,0004)**	PhotometricInterpretation	**(0028,1051)**	WindowWidth
**(0028,0010)**	Rows	**(0028,1052)**	RescaleIntercept
**(0028,0011)**	Columns	**(0028,1053)**	RescaleSlope
**(0028,0030)**	PixelSpacing	**(0028,1054)**	RescaleType
**(0028,0100)**	BitsAllocated	**(7FE0,0010)**	PixelData

**Table 3.** The Number of Images in the Training and Competition Data Sets Is Given

	No Evidence of Stroke or Chronic Ischemic Findings (May Or May Not Contain Other Pathologies)	Hyperacute/Acute Ischemic Stroke Findings	Hemorrhagic Stroke Findings	Both Hemorrhagic Stroke and Hyperacute/Acute Ischemic Stroke Findings or with Pathologies in Differential Diagnosis	Total
Training	4427	1131	1093	-	6651
Competition stage 1	130	35	35	-	200
Competition stage 2	-	48	47	5	100

**Table 4.** Shared Data-Files Folder and Properties Are Demonstrated

**Filename**	**Description**	**Matrix**	**Bits/ Channels**	**Format**	**Sample**
./DICOM/{imageid}.dcm	Anoymized DICOM image	512 × 512	16/1	DICOM	
./PNG/{imageid}.png	Image rendered for display	512 × 512	8/4	PNG	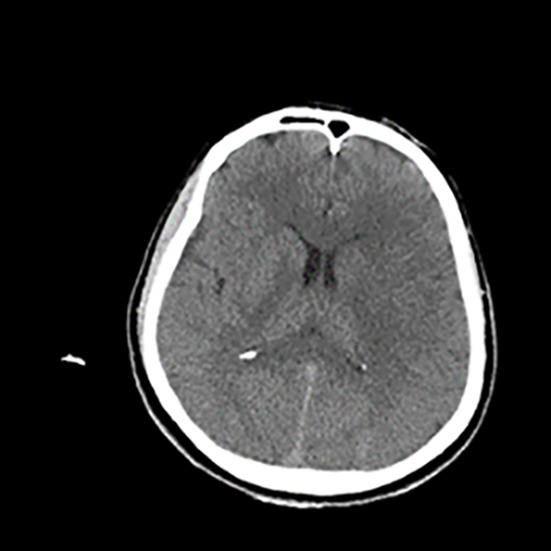
./MASK/{imageid}.png	Mask image consisting of 3 possible pixel values (0 : background, 1 : ischemic, 2 : hemorrhagic)	512 × 512	8/1	PNG	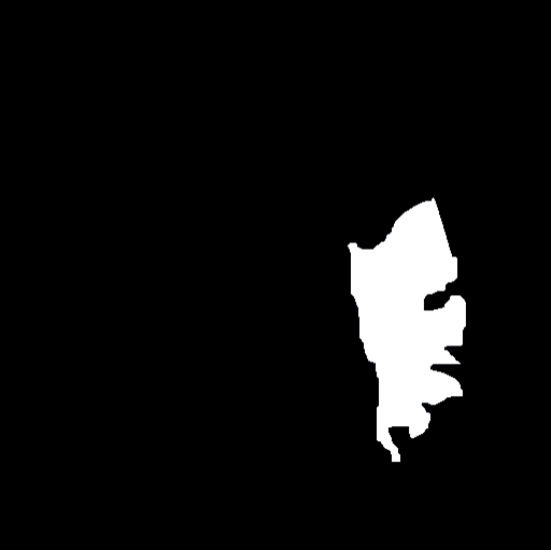
./OVERLAY/{imageid}.png	Original image blended with the mask image using a semi-transparent color. Boundaries of the mask are drawn with blue brush for ischemic and green brush for hemorrhagic lesions.	512 × 512	8/4	PNG	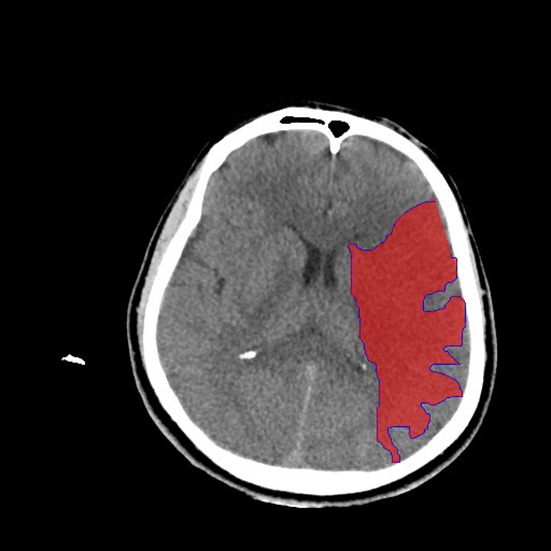

DICOM, digital imaging and communications in medicine; PNG, portable network graphics.

**Table 5.** Demographics of Team Members

**Gender**	**Total (n = 570)**	**Finalist (n = 225)**
Male, n (%)	421 (73.9)	174 (77.3)
Female, n (%)	149 (26.1)	51 (22.7)
Age, mean ± SD, min.-max., years	26.06 ± 7.48, 15-55	27.09 ± 8.49, 15-55
**Educational status**		
PhD student, n (%)	77 (13.5)	35 (15.6)
Postgraduate, n (%)	66 (11.6)	23 (10.2)
Graduate, n (%)	83 (14.6)	39 (17.3)
Undergraduate, n (%)	293 (51.4)	108 (48)
Vocational school, n (%)	2 (0.3)	0 (0)
High school student, n (%)	49 (8.6)	20 (8.9)

SD, standard deviation.
